# Assessment of patient safety culture in primary health care in Muscat, Oman: a questionnaire -based survey

**DOI:** 10.1186/s12875-019-0937-4

**Published:** 2019-04-05

**Authors:** Muna Habib AL Lawati, Stephanie D. Short, Nadia Noor Abdulhadi, Sathiya Murthi Panchatcharam, Sarah Dennis

**Affiliations:** 1 Discipline of Physiotherapy, Faculty of Health Sciences, The University of Sydney, Sydney, Australia; 2 Ministry of Health, P.O.Box, 626, PC 117 Wadi Al Kabir, Muscat Oman; 3 Statistical Department, Oman Medical Specialty Board, Way # 4443, Bld. 18, Block 244, Plot 404, North Azaiba, Muscat Oman; 4 South Western Sydney Local Health District, Liverpool, NSW 2171 Australia; 5 Ingham Institute of Applied Medical Research, 1 Campbell Street, Liverpool, NSW 2171 Australia

**Keywords:** Patient safety, Safety culture, Primary health care, medical errors, Oman

## Abstract

**Background:**

Patient safety is a universal issue which affects countries at all stages of health system development. Patient safety research in primary care reveals that globally millions of people suffer disabilities, injuries, or death due to unsafe medical practices. This study aims to explore the understanding of frontline primary health care professionals regarding patient safety culture in health care facilities in Oman.

**Methods:**

A questionnaire–based survey was conducted using a validated Hospital Survey of Patient Safety Culture tool. Invitations were sent to all 198 health professionals from each occupational category from each primary care center in Muscat, Oman.

**Results:**

The total number of respondents was 186 participants out of 198 (response rate: 94%). Overall, the staff had a strong sense of teamwork within the units (85%), they reported organization learning for continuous improvement (84%) and teamwork across the units (82%). However, the four dimensions which received the lowest scores were related to communication problems between the staff (23%), non-punitive response to errors (27%), frequency of event reporting (40%), and errors occurring when transferring patients to higher levels of health care during handoffs and transitions (46%).

**Conclusions:**

Overall, the participants rated patient safety in the primary health care setting as excellent or very good and the perception of patient safety was moderately positive. The core areas of strength were teamwork within the units with positivity and organization learning and continuous improvement. The weaknesses were non-punitive response to errors, inadequate staffing and hand offs and transition. The results of this study will provide policy makers and health care professionals with a detailed understanding of the current patient safety culture in primary care in Muscat, Oman. The results will be used by the Ministry of Health to inform policy and strategies to strengthen patient safety within primary health care in Oman.

**Electronic supplementary material:**

The online version of this article (10.1186/s12875-019-0937-4) contains supplementary material, which is available to authorized users.

## Background

Unsafe medical practices lead to disabilities, injuries and death each year to millions of patients worldwide [1] . The World Health Organization (WHO) defines patient safety as “the prevention of errors and adverse effects to patients associated with health care” and “to do no harm to patients” [2]. The aim is to reduce the risk of unnecessary harm associated with health care to an acceptable minimum. The acceptable minimum is context specific, based on current knowledge, resources available and balanced against the risk of alternative treatment options [3].

“To Err is Human: Building a Safer Health System” was published in 1999 by the Institute of Medicine (IOM), it highlighted that safety was an important concern. Patient safety in hospitals has received more attention as compared to primary care [4]. However, in most healthcare systems the majority of patient consultations take place in primary care and many of the incidents identified in hospitals may have originated in primary care, making the need for primary care patient safety research even more important.

There is no single standard to identify patient safety incidents in primary care [5]. The literature suggests that 24 - 85% of all harmful events occurring in primary are preventable [6]. This varies with research suggesting that in high income countries 50% of harm in primary care is preventable increasing to 60% in low income countries [7]. Attaining a culture of safety is a vital first step and requires an understanding of the values, beliefs, and norms about what is significant in an organizations, and what attitudes and behaviors applicable to patient safety are anticipated [8]. Organizations with a positive safety culture are characterized by communications founded on mutual trust, shared perceptions of the importance of safety, and by confidence in the efficacy of preventive measures [8].

There has been little research on patient safety published from Oman and other Gulf Cooperation Council (GCC) countries. There has been one published study in patient safety culture in primary healthcare in Kuwait [9] and three studies in the Eastern Mediterranean Region (EMRO) [9–11]. The Ministry of Health (MOH) in Oman has been working for many years at different levels to improve the quality and safety of health care services. A national working team was established in 2012 to develop a national action plan, guidelines, and mechanisms for monitoring and follow-up of aspects of patient safety in Oman [12]. Research into patient safety was identified as an important priority by the MOH, Oman, in its 2050 vision [13]. This study was conducted in this fertile context for research. Muscat is the capital of Oman, and the pilot region for new proposals and initiatives from the MOH since it covers a wide area ranging from a modern cosmopolitan population to remote areas where access to health care is limited [14]. The Sultanate of Oman is a high-income Arab country. It has experienced a rapid economic and social transformation since the 1970s which has resulted in better quality living standards [15]. The total population of Oman is 4.4 million and a third of the total population of Oman live in the capital city Muscat [16]. Primary health care centers (PHCCs) are the entry point for most patients with different health conditions in the publicly-funded Omani healthcare system.

The aim of this study was to explore the understanding of primary health care professionals regarding patient safety culture in primary health care facilities in Oman, in order to establish a baseline for the strengthening of patient safety in primary health care in Oman.

## Methods

A cross-sectional survey was undertaken to assess the patient safety culture in primary health care in Muscat, the capital of Oman between January and June 2016. A validated self-administered questionnaire, the Hospital Survey on Patient Safety Culture (HSOPSC) developed by the Agency of healthcare Research (AHRQ) [17] was used to assess the current patient safety culture among healthcare professionals in primary care [18].

The HSOPSC has been used in studies from the USA, UK and Europe in the hospital setting [19, 20]. It has also been used in the hospital setting in in the Middle East including Kuwait [9], Turkey [21], Iran [10] and Yemen [11]. It has been adapted and validated for primary care use in Portugal [22] and Switzerland [23]. It is a valid and a reliable tool developed on the basis of previous literature, cognitive tests and factor analysis. Safety culture variations have been reported across healthcare facilities, departments and occupational categories of healthcare workers in North America, Europe, Asia, and the Middle East [24]. The instrument includes 42 items grouped into 12 composite measures. It includes also two questions that ask respondents to provide an overall grade on patient safety for their work and to indicate the number of events they reported over the past 12 months. The scale used is a five-point Likert scale which ranges from ‘Strongly disagree’ to ‘Strongly agree’, or from ‘Never’ to ‘Always’ when relevant. A global safety grade between ‘poor’ and ‘excellent’ and the numbers of reported incidents in the past 12 months were also assessed.

### Pre-test/pilot study

Pretesting of the questionnaire was conducted with six frontline health care professionals who were family physicians, nurses and policy makers working in the MOH, Oman. The questionnaire was not translated into Arabic because all health professionals in Oman speak English. This was followed by modifications of the questionnaire for the primary health care setting in Oman. The term hospital was replaced by primary health care centers and the name of the districts, locally known as (wilayats) were included following their feedback from the primary health care professionals and the policy makers (Additional file 1).

Primary health care centers in Muscat, the capital of Oman, were included if they had the following services: general practice, nursing care and pharmacy and these services were functional seven days a week in addition to services of laboratory, radiology and dentistry care provided five days a week, excluding weekends. A systematic random sampling scheme was used in each primary health center, with assistance from an administrator, to select a sample of 10% condition sampling from each health professional category which represented the primary health care workforce. Health professionals were eligible for inclusion if they were full-time frontline health care professionals working in primary health care centers in the Muscat region (There are no part-time health care professionals working in the Ministry of Health in Oman). The health professions included doctors, nurses, dentists, pharmacists, radiographers, and laboratory technicians.

The selected health professionals were invited by an intermediary, a head nurse, and the principal investigator (MAL) explained the importance of the survey and its potential impact on safety in primary health care in addition to importance of their participation. The survey was given to the selected participants in a sealed envelope during working hours. Surveys were collected in a sealed envelope two to three days after distribution. A total of 198 health professionals from primary health care centers in the Muscat region were invited to take part in the survey and verbal consent was taken from the participant. All the sealed envelopes were collected from each health center by the intermediary. The participants were advised not to discuss the questionnaire with each other to avoid peer influence. The intermediary was not included as a participant.

### Sample size

The population of the survey was health care professionals in primary health care in the Muscat governorate (*N* = 1984). The total number of health care professionals working in primary health care in Muscat included in the study (*N* = 1164) this population reflects the 22 out of 28 health centers which were included in the study. The 22 out of 28 healthcare centers were selected on the basis of services which includes general practice, nursing care and pharmacy these services are functional 7 times a week in addition to services such as laboratory, X ray services and dentistry care provided 5 days a week excluding weekends. We aimed to survey a representative sample of 10% of the total from each occupational category. From each center we sampled the following: Nurses (*n* = 3), Physicians (*n* = 2), Radiographers (*n* = 1), Laboratory technicians (n = 1), Dentists (n = 1) and Pharmacists (n = 1) (9 in total from each center). Thus, the sample size for this study was (9 × 22 = 198) health professionals from the selected primary care centers.

### Data management

The principal investigator (MAL) entered the data into the University of Sydney’s REDCAP database [25]. No identifying information was obtained from the participants, confidentially and anonymity were assured and maintained.

### Statistical analysis

Data were analyzed using SPSS 22 statistical software (IBM Corp. Released 2013. IBM SPSS Statistics for Windows, Version 22.0. Armonk, NY: IBM Corp). Descriptive statistics were used to explore the characteristics of the respondents. Calculation of the composite frequency for the twelve patient safety dimensions measured by the HSOPSC data collection tool was conducted as per the user guidelines published by AHRQ [26]. Items were worded in negative and positive directions. The composite frequency was calculated by dividing the total number of positive responses of all the items constituting a dimension (numerator) by the total number of the responses to all the items of that dimension excluding missing responses (denominator) times 100. The resulting number represents a positive response on that specific dimension [26]. The responses for each item in the dimensions were strongly disagree, disagree, neither, agree and strongly agree, and from ‘never’ to ‘always’ when relevant. Positive responses were considered whenever strongly agree and agree where chosen. Composite frequencies of the total percentage of the positive responses of each dimension were calculated in addition to each item and primary health care center.

### Ethical consideration

The study protocol was approved by the Research and Ethical Review and Approval Committee at the Centre of Research and Studies in the Ministry of Health on 2nd February 2016, Muscat, Oman. A permission letter was further sent to all the health centers and verbal informed consent was obtained from each participant. *See* Additional file 2* for the research ethics approval letter.*

## Results

### Demographic data

Out of the 28 operating primary health care centers in the Muscat area, 22 met the inclusion criteria. There were 186 completed questionnaires from the 198 health professionals invited to participate (response rate of 94%). The demographic characteristics of the participating health professionals are detailed in Table 1.

**Table 1:** Demographic characteristics of the participating health professionals

Health Professional Characteristics	Number (%) (*n* = 186)
Gender	
Female	176 (95%)
Male	10 (5%)
Professional background	
Nurses	61 (33%)
Physicians	42 (23%)
Radiographers	22 (12%)
Laboratory technicians	22 (12%)
Dentists	20 (11%)
Pharmacists	18 (10%)
Age group	
20–30 years	59 (32%)
31–40 years	102 (55%)
41–50 years	23 (12%)
51–60 years	2 (1%)
Number of years working in health center	
< 1 year	23 (13%)
1–5 years	98 (53%)
6–10 years	41 (22%)
11–15 years	15 (9%)
> = 16 years	6 (3%)
Number (%) who had worked in another country	35 (19%)

Overall, 74% (139/186) of the staff who participated in the survey graded patient safety as excellent or very good, and 63% (116/186) of staff in the health centers had not reported any events in the past 12 months and only a third (60/186) had reported one to five events, see Table 2.

- Unit level (Department level)

**Table 2:** Patient safety as graded by the staff and the number of staff reporting events in the last 12 months

Variables		n = 186	%
Patient Safety Grade	Excellent	38	20
	Very good	101	54
	Acceptable	46	25
	Poor	1	0.5
Event Reporting	No event reports	116	63
	1–5	60	33
	6–20	6	3
	≥21	3	2

The average positive response for all the dimensions at the unit level was 59%. Table 3 provides details showing the percentage of the positive responses for the twelve dimensions within the primary health care centers. Overall, the staff had a strong sense of teamwork within units (85%), organization learning for continuous improvement (84%) and teamwork across the units (82%). Dimensions which had less than 50% of average positive responses were staffing (23%) non- punitive response to errors (27%) and frequency of events reporting (40%). Under non – punitive response to errors 20% gave response in terms of worrying that their mistakes would be kept in their personal files. A third (32%) responded that if an error was reported, it would be the staff member who was written up and not the error, see Table 4**.**

b. Primary health care facility level

**Table 3:** Dimensions with positive responses for the twelve dimensions within the primary health care centers

Dimensions	Dimension’s positivity
Safety culture dimension at the unit level	
Teamwork within Units	85%
Supervisor/Managers expectations and actions promoting patient safety	59%
Organization learning continuous improvement	84%
Feedback and communication about error	65%
Communication openness	68%
Staffing	23%
Non-punitive response to error	27%
Safety culture dimensions at the primary health care facility level	
Hand-offs and transitions	46%
Teamwork across units	82%
Management support for patient safety	75%
Outcome measures of patient safety culture	
Frequency of error reporting	40%
Overall perception of patient safety	55%

**Table 4:** Description of safety culture dimension at the unit level

Work area / Unit	Number (%) of positive responses	Total responses
Teamwork within Units (Dimension’s positivity = 85%)		
People support one another in this unit	171 (92)	186
When a lot of work needs to be done quickly, we work together as a team to get the work done	164 (88)	186
In this unit, people treat each other with respect	166 (89)	186
When one area in this unit gets busy, others help out	129 (69)	185
Supervisor/Managers expectations and actions promoting patient safety (Dimension’s positivity = 59%)		
My supervisor/manger says a good word when he/she sees a job done according to established patient safety procedures	148 (80)	185
My supervisor /manger seriously considers staff suggestions for improving patient’s safety	161 (87)	184
Whenever pressure builds up, my supervisor/manger wants us to work faster, even if it means taking shortcuts	80 (43)	186
My supervisor/manger overlooks patient safety problems that happen over and over	44 (24)	180
Organization learning continuous improvement (Dimension’s positivity = 84%)		
We are actively doing things to improve patient safety	178 (96)	186
Mistakes have led to positive changes here	147 (79)	186
After we make changes to improve patient safety, we evaluate their effectiveness	143 (77)	186
Feedback and communication about error (Dimension’s positivity = 65%)		
We are given feedback about changes put into place based on event reports	91 (49)	185
We are informed about errors that happen in this center	124 (67)	186
In this center, we discuss ways to prevent errors from happening again	144 (77)	184
Communication openness (Dimension’s positivity = 68%)		
Staff will freely speak up if they see something that may negatively affect patient care.	144 (77)	186
Staff feel free to question the decisions or actions of those with more authority	112 (60)	186
Staff are afraid to ask questions when something does not seem right	121 (65)	185
Staffing (Dimension’s positivity = 23%)		
We have enough staff to handle the workload	76 (41)	186
Staff in this unit work longer hours than is best for patient care	21 (11)	186
We work in “crisis mode” trying to do too much, too quickly	31 (17)	186
Non-punitive response to error (Dimension’s positivity = 27%)		
Staff feel like their mistakes are held against them	52 (28)	186
When an event is reported, it feels like the person is being written up, not the problem	59 (32)	186
Staff worry that mistakes they make are kept in their personnel file	38 (20)	182

Table 5 presents the results at the health care facility level. At the primary health care level center, the handoff and transitions dimension had a positivity of 46%. The survey revealed that problems and errors occurred when transferring patients to secondary care, as only 19% gave positive responses.

c. Outcome measures of patient safety culture

**Table 5:** Description of safety culture dimensions at the primary health care facility level

Work area / Unit	Positive responses N (%)	No. of Total responses
Hand-offs and transitions (Dimension’s positivity = 46%)		
Things “fall between the cracks” when transferring patient to and from	173 (93)	179
Within health centers	95 (51)	179
Secondary care	36 (19)	182
Tertiary care	161 (87)	186
Important patient care information is often lost during shift changes	129 (69)	185
Problems often occur in the exchange of information across sections in the health center.	162 (87)	184
Shift changes are problematic for patients in this health center	104 (57)	183
Teamwork across units (Dimension’s positivity = 82%)		
There is good cooperation among health center sections that need to work together	120 (65)	186
Health center sections work well together to provide the best care for patients	104 (56)	186
The clinics do not coordinate well each other	137 (74)	181
Management support for patient safety (Dimension’s positivity = 75%)		
The center management seems interested in patient safety only after an adverse event happens	175 (94)	186
The health center management provides a work climate that promotes patient safety	156 (84)	185
The actions of the center management show that patient safety is a top priority	97 (52)	186

As highlighted in Table 6, the frequency of error reporting was the third least-scored patient safety dimension among the primary health care staff with an overall positive response of 40%. All the items scored less than 50% positive responses. There was 34% positive response to the statement about when a mistake was made by a staff member but had no potential to harm the patient. The overall perception of patient safety was moderate (dimensions positivity is 55%) as 59% of staff reported that they did not sacrifice patient safety to get more work done, 64% of participants agreed that their procedures and systems were good at preventing errors from happening. About 44% of participants responded that more serious mistakes did not happen and 51% reported positively that there were no safety problems in the unit.

**Table 6:** Outcome measures of patient safety culture

Outcome measures	Positive responses N (%)	No. of Total responses
Frequency of error reporting (Dimension’s positivity 40%)		
When a mistake is made, but is caught and corrected before affecting the patient, how often is this reported?	72 (39)	186
When a mistake is made, but has no potential to harm the patient, how often is this reported?	63 (34)	186
When a mistake is made that could harm the patient, but does not, how often is this reported?	91 (49)	186
Overall perception of patient safety (Dimension’s positivity = 55%)		
Patient safety is never sacrificed to get more work done	110 (59)	185
Our procedures and systems are good at preventing errors from happening	119 (64)	185
It is just by chance that more serious mistakes don’t happen around here	82 (44)	186
We have patient safety problems in this unit	95 (51)	186

## Discussion

This study is the first to measure and analyze patient safety culture in the primary health care setting in Muscat, Oman. The response rate was high, and responses were obtained from a range of frontline primary health care professionals. Overall, the participants rated patient safety in the primary health care setting as excellent or very good and the perception of patient safety was moderately positive. At the unit level, there was a strong sense of organizational teamwork and processes to support continuous improvement. However, staffing and non-punitive response to errors were a concern within the units and at the health center level hand offs and transitions to other health institutions and hospitals were an issue and overall, the response to errors was poor.

The findings from the primary care setting are comparable to a study conducted with heath care professionals in secondary and tertiary care hospitals in the northern region of Oman (*n* = 368) [8]. In that study, the overall positive response was 59% and the dimensions which rated lowest were also non-punitive response to error (25%), staffing (33%) and hand offs and transitions (44%). The frequency of error reporting scored higher (65%) because in the hospitals there was an established system for error reporting in contrast to the primary health care. In spite of this, the culture of blame still existed, and health care professionals were fearful of punishment or job loss for reporting errors.

Studies conducted in other Middle Eastern countries have reported similar results on error reporting. Whilst the positive results may seem low, they are actually high compared to a similar study conducted in 12 primary health care centers with 180 staff members surveyed in Turkey where the frequency of error reporting was 12% [21]. A similar study in Kuwait [9] surveyed 223 health professionals in 22 centers, they reported 24% non-punitive response to errors compared to 27% in the current Omani study, which is quite similar possibly due to relatively high positivity of the organizational learning 74% in Kuwait and 79% in current Omani study. Health center hand offs and transitions showed similar results to Kuwait, indicating there were issues with safe continuity of care when patients were transferred to secondary and tertiary care. Additionally, teamwork across the units was scored equally between the Omani and the Kuwaiti primary health care. The area with the lowest positive score in this study was inadequate staffing (23%). This was low compared to results from Iran (38%), Kuwait (41%), Turkey (49%) and Yemen (50%). A possible reason for this is that the number of staffs in each health center in Muscat is standardized. However, due to population movements to the suburbs there have been increases in the population of the catchment area corresponding to the health centers placing more demands on the available staff. Furthermore, in the last three years no new health centers have been built by the MOH in Muscat.

The MOH has developed a long-term vision for the development and strengthening of the health care system in Oman. In 2012, “Vision 2050” was issued and distributed. Vision 2050 comprehensively examined the health system functions, namely the political; economic; social; technological; environmental; and legal environments (PESTEL analysis) [13]. Furthermore, Oman’s Quality Assurance and Patient Safety vision for 2050 specified that “the quality and patient safety health care in Oman will be recognized internationally as a top healthcare system that responds to community needs through community empowerment and partnership and provides quality, sustainable and innovative health care services through committed competent and efficient staff” [13]. The results of this survey will be used to inform progress towards realization of these objectives in patient safety in primary health care in Oman.

The culture of an organization needs to be assessed first, before effective change in an organization can occur [13]. Falling under the umbrella of “vision 2050” are the national five years’ health development plans. Outcome of this study is expected to be taken into consideration for the next five years (2020–2025), develop plan for establishing of a national survey to assess the perception of patient safety in primary care across all the regions of Oman. The results of this study will assist the Ministry of Health to implement key strategies for improvement. Furthermore, the Ministry of Health will further take necessary steps for partnership with other Ministries if required.

This survey had a few limitations that need to be considered. The language of the survey was English, even though English was not the native language of all participants, but is the language used among health care professionals and most of the medical college and technical schools teach in English.

The data were analyzed by calculating the percentage of positive responses but averaging individual means has been found to give a better precision [24]. Some critics might recommend using the Safety Attitudes Questionnaire to assess patient safety culture but it has been noted that the HSPSC has similar psychometric properties [27]. The HSPSC has in addition similar psychometric properties in various population settings, including the Arabic speaking one [28] however, they were not tested in this study. Another possible limitation is that the Medical Office Survey on Patient Safety Culture (MOSOPSC) tool which was designed for use in primary care and used in the Portuguese Primary Healthcare [22] could have been used however the aim was to use a tool which had previously been used in the Gulf region.

## Conclusions

This survey of patient safety culture in primary health care in Muscat, Oman, has identified that the core areas of strength are teamwork within the units and organization learning and continuous improvement. Areas which require improvement are non-punitive response to errors, inadequate staffing and hand offs and transition. The findings from this study will provide policy makers and health care professionals with a detailed understanding of the current patient safety culture in Muscat, Oman as a foundation for improvements in patient safety, led by the Ministry of Health in Oman. A well-designed national patient safety initiative is required which should be integrated into primary health care center policies and in the upcoming five-year organizational plan which aims to improve communication openness and to establish an automated incidence reporting system. Staffing levels and handoffs and transitions also demand closer attention.

## Additional files

Additional file 1: The questionnaire. (PDF 872 kb)

Additional file 2: Approval letter. (PDF 32 kb)

## Abbreviations

AHRQ: Agency of healthcare Research and Quality
EMRO: Eastern Mediterranean Region
GCC: Gulf Cooperation Council Countries
HSOPSC: Hospital Survey of Patient Safety Culture tool
MOH: Ministry of Health
WHO: World Health Organization
