# Supplementary material for: Assessment of patient safety culture in primary health care in Muscat, Oman: a questionnaire -based survey
Source: BMC Fam Pract. 2019 Apr 5;20:50. doi: 10.1186/s12875-019-0937-4 (PMC6449986; doi:10.1186/s12875-019-0937-4)
Supplement: Supplementary file 1 — The questionnaire. (PDF 872 kb) [file 12875_2019_937_MOESM1_ESM.pdf]

Information sheet for safety culture in primary health care centres in Muscat region.

Title of Research Study:

"*Patient Safety culture Survey*"

INFORMATION SHEET FOR PARTICIPANTS

Dear Client

You are invited to take part in a research study to examine the safety culture of primary care in the Muscat Region. You will be requested to fill out a questionnaire which is self-administered.
Before you decide whether to take part in the study it is important that you understand what the research is for and what you will be asked to do.

Please take time to read the following information and discuss it with others if you wish. It is up to you to decide whether to take part or not. If you decide to take part, you will be given this information sheet to keep.

The purpose of the research study is to explore patient safety culture in primary care centres in Muscat.

The information gained from this research will be used to make recommendations for best practice and will offer insights to plan policies and guidelines for best practices in primary care in Oman. The questionnaire is confidential, and no identity will be revealed. This research is part of a PhD thesis at University of Sydney, Australia.

Please do not hesitate to contact me if you need further information

Thanking you in anticipation,

Yours sincerely,

Dr Muna Habib AL Lawati
99467686

THE UNIVERSITY OF SYDNEY

Ministry of Health
Sultanate of Oman

Directorate General of Health Services

Governorate of Muscat

Quality Management Section

Patient Safety Culture Survey

Instructions:

This survey asks for your opinion about patient safety issues, medical error, and event reporting in your health center and will take about 15 to 20 minutes to complete.

All data collected is anonymous.

An "***event***" is defined as any type of error, mistake, incident, accident, or deviation, regardless of whether or not it results in patient harm.

"***Patient safety***" is defined as the avoidance and prevention of patient injuries or adverse events resulting from the process of health care delivery.

Section A:

Please indicate your agreement or disagreement with the following statements about your workplace:

**S.N**	**Statement**	**Strongly disagree**	**Disagree**	**Neither**	**Agree**	**Strongly Agree**
**1**	People support one another in the health center					
**2**	We have enough staff to handle the workload					
**3**	When a lot of work needs to be done quickly, we work together as a team to get the work done					
**4**	In this health center, people treat each other with respect					

Adapted from Agency for Healthcare Research and Quality(http://www.ahrq.gov/qual/hospculture/)

**5**	Staff work longer hours in this health center for best patient care					
**6**	We are actively doing things to improve patient safety					
**7**	Staff feel like their mistakes are held against them					
**8**	Mistakes have led to positive changes here					
**9**	It is just by chance that more serious mistakes don't happen					
**10**	When one area in this health center gets really busy, others help out					
**11**	When an event is reported, it feels like the person is being written up, not the problem					
**12**	After we make changes to improve patient safety, we evaluate their effectiveness					
**13**	We work in "crisis mode" trying to do much, too quickly					
**14**	Patient safety is never sacrificed to get more work done					
**15**	Staff worry that mistakes they make are kept in their personal file					
**16**	We have patient safety problems in this center					
**17**	Our procedures and systems are good at preventing errors from happening					

Section B: Supervision / management

Please indicate your agreement or disagreement with the following statements about your immediate supervisor/manager or person to whom you directly report.

S.N	Statement	Strongly disagree	Disagree	Neither	Agree	Strongly Agree
**1**	My supervisor/manger says a good word when he/she sees a job done according to established patient safety procedures					

2	My supervisor /manger seriously considers staff suggestions for improving patients safety					
3	Whenever pressure builds up, my supervisor/manger wants us to work faster, even if it means taking shortcuts					
4	My supervisor/manger overlooks patient safety problems that happen over and over					

Section C: Communication

How often do the following things happen in your work area?

S.N	Statement	Never	Rarely	Sometimes	Most of the time	Always
1	We are given feedback about changes put into place based on event reports					
2	Staff will freely speak up if they see something that may negatively affect patient care.					
3	We are informed about errors that happen in this center					
4	Staff feel free to question the decisions or actions of those with more authority					
5	In this center, we discuss ways to prevent errors from happening again					
6	Staff are afraid to ask questions when something does not seem right					

Section D: Frequency of Events Reported

In your health center, when the following mistakes happen, how often are they reported?

S.N	Statement	Never	Rarely	Sometimes	Most of the time	Always
1	When an error is made, but is *caught and corrected before affecting the patient*, how often is this reported?					
2	When an error is made, but *has no potential to harm the patient*, how often is this reported?					
3	When an error is made that *could harm the patient, but does not*, how often is this reported?					

Section E:

Please give your health center an overall grade on patient safety.

A	B	C	D
Excellent	Very good	Acceptable	Poor

Section F: Your Health Center

Please indicate your agreement or disagreement with the following statements about your health center.

Think about ***your*** health center

S.N	Statement	Strongly disagree	Disagree	Neither	Agree	Strongly Agree
1	The health center management provides a work climate that promotes patient safety					
2	The clinics do not coordinate well each other					
3	Things "fall between the cracks" when transferring patient to and from	(Please tick)				
	a) within health centers					
	b) secondary care					
	c) tertiary care					
4	There is good cooperation among health center sections that need to work together					
5	Important patient care information is often lost during shift changes					
6	It is often unpleasant to work with staff from other health centers					
7	Problems often occur in the exchange of information across sections in the health center.					
8	The actions of the center management show that patient safety is a top priority					
9	The center management seems interested in patient safety only after an adverse event happens					

Adapted from Agency for Healthcare Research and Quality(http://www.ahrq.gov/qual/hospculture/)

**10**	Health center sections work well together to provide the best care for patients					
**11**	Shift changes are problematic for patients in this health center					

Section G: Number of Events Reported:

1 In the past 12 months, how many event reports have you filled out ***and*** submitted?

**a**. No event reports		**d**.6 to 10 event reports	
**b**. 1 to 2 event reports		**e**.11 to 20 reports	
**c**.3 to 5 event reports		**f**. 21 event reports or more	

2 In the past 12 months, how many event reports have you filled out ***and not*** submitted?

**a**. No event reports		**d**.6 to 10 event reports	
**b**. 1 to 2 event reports		**e**.11 to 20 reports	
**c**.3 to 5 event reports		**f**. 21 event reports or more	

Section H: Background Information:

Please provide some information about you:

1. How long have you worked in this health center?

**a**. Less than 1 year		**d**.11 to 15 years	
**b**. 1 to 5 years		**e**.16 to 20 years	
**c**. 6 to 10 years		**f**. 21 years or more	

2. Typically how many hours per week do you work in this center?

**a**. Less than 20 hours per week		**d**. 60 to 79 hours per week	
**b**. 20 to 39 hours per week		**e**. 80 to 99 hours per week	
**c**. 39 to 59 hours per week			

3. What is the name of your Wilayat?

**a**. Al-Seeb		**d**. Quriyat	
**b.** Bousher		**e**. Muscat	
**c**. Al-Amerat		**f**. Mattrah	

4. What is your profession?

**a.** Doctor	
**b**. Nurse	
**c**. Dentists	
**d.** Pharmacist	
**e.** Laboratory technicians	
**f.** Radiographer	

5 Please specify your age group

**a**. 20-30	
**b.** 31-40	
**c**. 41-50	
d. 51-60	
e. 61+	

6 Please specify your gender

**a.** Male	
**b.** Female	

Thank you for taking the time to complete this survey
