# Supplementary material for: Assessment of patient safety culture in primary health care in Muscat, Oman: a questionnaire -based survey
Source: BMC Fam Pract. 2019 Apr 5;20:50. doi: 10.1186/s12875-019-0937-4 (PMC6449986; doi:10.1186/s12875-019-0937-4)
Supplement: Supplementary file 2 — Approval letter. (PDF 32 kb) [file 12875_2019_937_MOESM2_ESM.pdf]

Sultanate of Oman
Ministry of Health
Directorate General of Planning
and Studies

سلطنة عمان
وزارة الصحة
المديرية العامة للتخطيط
والدراسات

Ref. : MH/DGP/R&S/PROPOSAL_ APPROVED/3/2016 — الرقم: ....

Date : 2.2.2016 — التاريخ: ....

الموافق: ....

Muna Habib Al Lawati
Principal Investigator

Study Title: **"Patient safety culture assessment in primary care in Muscat, Oman".**

After compliments

We are pleased to inform you that your research proposal **"Patient safety culture assessment in primary care in Muscat, Oman"** has been approved by Research and Ethical Review & Approve Committee, Ministry of Health.

Regards,

Dr. Ahmed Mohamed Al Qasmi
Director General of Planning and Studies
Chairman, Research and Ethical Review and Approve Committee
Ministry of Health, Sultanate of Oman.

Cc
Day file
